# Pydiflumetofen Co-Formulated with Prothioconazole: A Novel Fungicide for Fusarium Head Blight and Deoxynivalenol Control

**DOI:** 10.3390/toxins14010034

**Published:** 2022-01-03

**Authors:** Simon G. Edwards

**Affiliations:** Crop and Environmental Sciences, Harper Adams University, Newport TF10 8NB, UK; sedwards@harper-adams.ac.uk

**Keywords:** *Fusarium graminearum*, deoxynivalenol, pydiflumetofen, ADEPIDYN^™^, prothioconazole, wheat

## Abstract

Fusarium head blight (FHB) is an important disease of small grain cereals worldwide, resulting in reduced yield and quality as well as the contamination of harvested grains with mycotoxins. The key mycotoxin of concern is deoxynivalenol (DON), which has legislative and advisory limits in numerous countries. Cereal growers have a number of control options for FHB including rotation, cultivation, and varietal resistance; however, growers are still reliant on fungicides applied at flowering as part of an IPM program. Fungicides currently available to control FHB are largely restricted to triazole chemistry. This study conducted three field experiments to compare a new co-formulation of pydiflumetofen (a succinate dehydrogenase inhibitor (SDHI) with the tradename ADEPIDYN^™^) and prothioconazole (a triazole) against current standard fungicides at various timings (flag leaf fully emerged, mid-head emergence, early flowering, and late flowering) for the control of FHB and DON. Overall, the co-formulation showed greater efficacy compared to either pydiflumetofen alone or current fungicide chemistry. This greater activity was demonstrated over a wide range of spray timings (flag leaf fully emerged to late flowering). The availability of an SDHI with good activity against FHB and the resulting DON contamination of harvested grain will give growers an additional tool within an IPM program that will provide a greater flexibility of spray application windows and reduce fungicide resistance selection pressure.

## 1. Introduction

Small grain cereals, and wheat in particular, are a key component of food and feed worldwide. Wheat is the second largest grain crop after maize, with a worldwide production of over 750 million tons [1]. The yield and quality of cereals is of paramount importance in feeding our ever growing global population [2]. Fusarium head blight (FHB) is a major impactor on the yield and quality of small grain cereals and wheat in particular, which is the most susceptible host species [3]. The disease is caused by a complex of fungal species that includes numerous *Fusarium* species and *Microdochium nivale* and *M. majus* [4,5], with epidemics occurring in all cereal growing region of the world [6]. All FHB pathogens can impact yield and various quality parameters, but only the *Fusarium* species are known to produce mycotoxins. Of the mycotoxins produced, the trichothecene, deoxynivalenol (DON) is the most commonly detected. Numerous entities have set advisory or regulatory limits for DON in cereals and cereal products intended for use as food or feed. The European Union sets a limit of 1.25 mg/kg in wheat intended for human consumption, lower limits for cereal products for direct consumption, and guideline limits for cereal intended for feed and compound animal feed in 2006 [7,8]. These limits are likely to be lowered in the near future to account for the combined toxicity of DON and its associated acetylated and glucosylated derivatives [9].

*Fusarium graminearum* is the most important FHB pathogen worldwide and is frequently associated with severe epidemics and high mycotoxin concentrations [10]. During epidemic years, the economic impact of FHB can be critical for the industry. For example, the 2015 US wheat harvest yield was reduced by $1.2 billion in value, along with the additional costs of downgraded premiums, testing, etc. [11].

FHB and resulting mycotoxins are best controlled by integrated pest management (IPM) incorporating several components including host resistance and cultural control (rotation and tillage) as well as fungicides. There are few fungicides with activity towards FHB and these are primarily demethylation inhibitors (DMI) of the triazole family, including propiconazole, tebuconazole, metconazole, and prothioconazole [12]. Studies tend to show that metconazole, prothioconazole, and a co-formulation of tebuconazole and prothioconazole have the greatest activity towards FHB and DON reduction [13].

FHB infection occurs at flowering and numerous studies have shown that fungicide application is most effective when applied from early to mid-flowering (Zadoks GS61-65 [14]) with studies showing a marked reduction in control of FHB when fungicides are applied earlier or later [12,15,16]. Fungicide applied at early flowering is the traditional third spray timing in UK wheat production and referred to as T3. A previous study at this laboratory found that less effective but still useful reductions of FHB and DON were achievable with earlier foliar applications of prothioconazole at the traditional first and second fungicide spray timing at stem extension (T1, GS32) and full flag leaf emergence (T2, GS39) [17].

Quinone outside inhibitor (QOI) fungicides have been shown to be ineffective and in some cases can increase mycotoxin levels [12,18]. Early succinate dehydrogenase inhibitors (SDHI) fungicides have been shown to be ineffective against FHB [19,20]. Recent publications have highlighted the efficacy of a new SDHI fungicide, ADEPIDYN™ (APN; ai pydiflumetofen, Syngenta Crop Protection AG, Basel, Switzerland) alone or in co-formulation with propiconazole (MIRAVIS ACE™) as having good activity towards FHB [21,22]. 

Pydiflumetofen is due to be launched to the European market in 2022 as a co-formulation with a different triazole, prothioconazole (APN + PTZ). As prothioconazole has greater efficacy against FHB than propiconazole, it was deemed useful to compare pydiflumetofen alone and in co-formulation with prothioconazole to prothioconazole alone (Proline™, Bayer AG, Monheim, Germany) and prothioconazole in co-formulation with two SDHI: bixafen and fluopyram (AscraXpro™, Bayer). As early results showed a high efficacy of pydiflumetofen products in FHB and DON control, it was deemed useful to identify if this extended the window of application around the typical application at flowering (T3) and if an earlier foliar application at full flag leaf emergence (T2) contributed to a greater reduction in FHB infection and DON.

## 2. Results

### 2.1. Experiment 1

**Figure 1:**
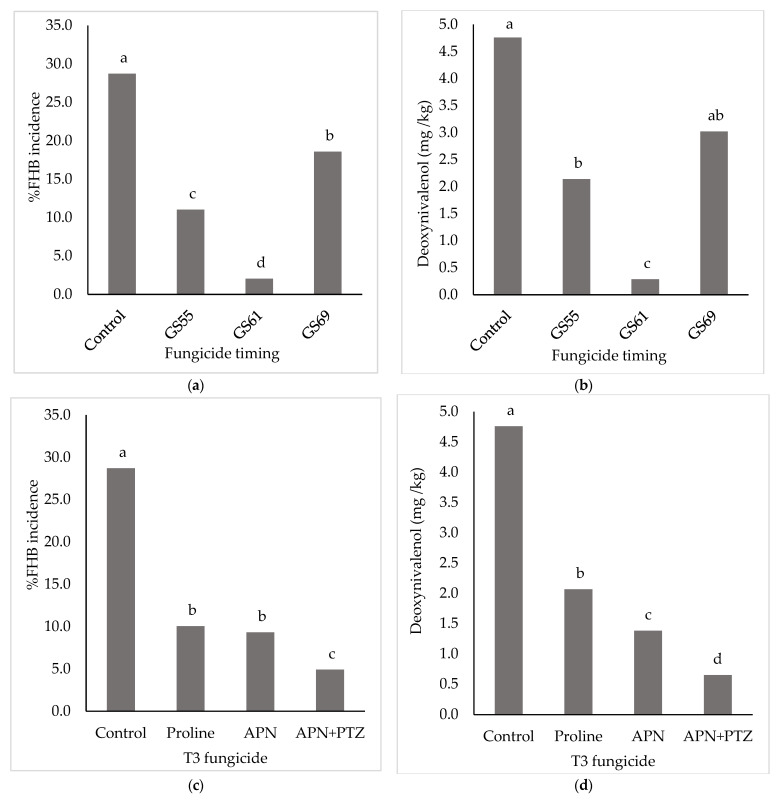
Percentage Fusarium head blight (FHB) incidence of winter wheat at late milky ripe (GS77) (**a**,**c**) and deoxynivalenol concentration of harvested grain (**b**,**d**) after application of fungicides at three timings around flowering (GS55, GS61, GS69) averaged across fungicides (**a**,**b**) and for three fungicides applied at maximum single field rate (**b**,**d**) averaged across application timings. Fungicides applied were Proline 275 (ai prothioconazole 275 g/L, 0.72 L/ha, Bayer) APN (A21857B; ai pydiflumetofen 62.5 g/L; 2.65 L/ha, Syngenta) and APN + PTZ (A20944K; ai pydiflumetofen 62.5 g/L plus prothioconazole 75 g/L; 2.65 L/ha, Syngenta). Bars with the same letter were not significantly different (Fisher LSD; *p* > 0.05).

There was no significant interaction between fungicide timing and fungicide product for either FHB disease incidence or deoxynivalenol concentration in harvested grains (*p* = 0.259 and 0.344, respectively). For both measured parameters, fungicide timing and product were very highly significant (*p* < 0.001). The untreated control had 29% incidence of FHB resulting in 4.75 mg/kg DON in harvested grains. All timings resulted in a significant (*p* < 0.05) reduction in %FHB, with the most effective reduction at GS61 (93%) and the least at GS69 (35%) (Figure 1a). Similar results for fungicide timing were obtained for DO; however, the overall mean for treatments applied at GS69 was not significantly different from the untreated control (Figure 1b). All fungicide products were applied at the maximum field rate and resulted in a significant reduction in %FHB incidence, with the greatest reduction (83%) achieved with the APN + PTZ co-formulation (Figure 1c). This provided significantly better control than APN or Proline alone, which were not significantly different from one another. A similar trend was seen for DON, with an 86% reduction with the APN + PTZ co-formulation (Figure 1d). However, for DON, APN alone also resulted in a significantly lower concentration compared to the Proline treatment. The APN + PTZ co-formulation at the optimum timing of GS61 gave a reduction of 96% for both FHB and DON.

The ANOVA contrast function (Genstat v20, VSN International Ltd., Hemel Hempstead, UK) was used to compare individual treatments at early and late timings to identify differences in fungicide performance away from the optimum timing. For FHB incidence, all fungicides significantly reduced disease compared to the untreated (*p* < 0.01) at the early application (GS55) but only the APN + PTZ co-formulation resulted in a significant reduction (*p* < 0.001) in FHB incidence compared to the untreated control when applied late (GS69). For DON, APN and the APN + PTZ co-formulation gave a significant (*p* = 0.035 and *p* < 0.001 respectively) reduction at the earlier timing, but for the later application only the APN + PTZ co-formulation gave a significant (*p* < 0.001) reduction compared to the untreated control.

### 2.2. Experiment 2 and 3

**Figure 2:**
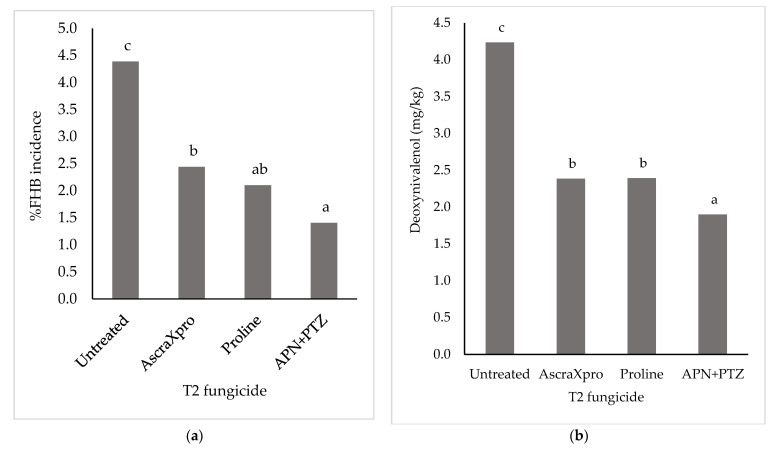

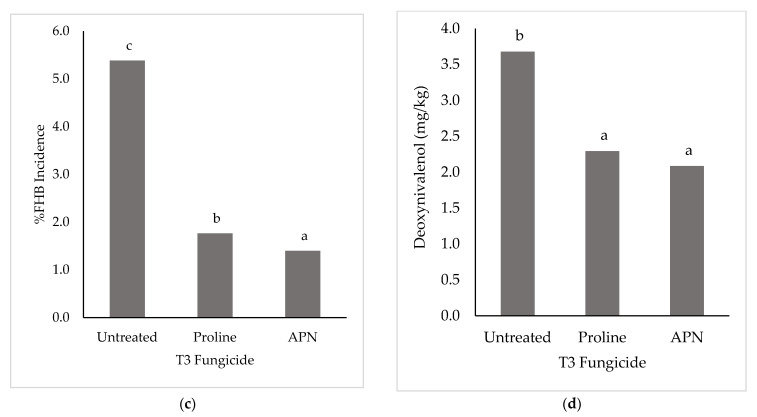
Percentage Fusarium head blight (FHB) incidence of winter wheat at late milky ripe (GS77) (**a**,**c**) and deoxynivalenol concentration of harvested grain (**b**,**d**) after application of fungicides at two timings; T2 (flag leaf fully emerged, GS39) (**a**,**b**) and T3 (early flowering, GS61) (**c**,**d**). Bars with the same letter were not significantly different (Fisher LSD; *p* > 0.05). T2 fungicides applied at 0.75 maximum single field rate were AscraXpro (ai bixafen 65 g/L, fluopyram 65 g/L and prothioconazole 130 g/L, 1.12 L/ha, Bayer), Proline 275 (ai prothioconazole 275 g /L, 0.54 L/ha, Bayer) and APN + PTZ co-formulation (A20944K; ai pydiflumetofen 62.5 g /L plus prothioconazole 75 g /L; 2.65 L/ha, Syngenta). T3 fungicides applied at 0.5 maximum single field rate were Proline 275 (ai prothioconazole 275 g /L, 0.54 L/ha, Bayer and APN (A21857B; ai pydiflumetofen 62.5 g /L; 2.65 L/ha, Syngenta). All fungicides at T2 were supplemented with Mirror (ai folpet 500 g /L; 1 L/ha). Results are the back-transformed means from Experiment 2 and 3.

The untreated control plots in Experiment 2 had a mean FHB incidence of 1.4% and DON concentration of harvested grains of 1.4 mg/kg. In Experiment 3 the mean levels in untreated controls plots were 48.4% FHB and 23 mg/kg DON respectively. Experiments 2 and 3 were of the same design and had equivalent variance, as such the results were combined in a single ANOVA. There was no significant interaction between the fungicides applied at T2 (GS39) and T3 (GS61) for either %FHB (*p* = 0.955) or DON (*p* = 0.993). There was a very highly significant (*p* < 0.001) reduction in %FHB incidence and DON concentration in harvested wheat grains from the application of fungicides at both T2 and T3. For T2, the co-formulation of APN + PTZ resulted in the greatest reduction in %FHB (68%) and DON (55%) and this reduction was significantly greater than AscraXpro for disease incidence and for both AscraXpro and Proline for DON (Figure 2a,b). There was no significant difference in the reduction in FHB or DON for AscraXpro compared to Proline.

At T3, APN resulted in a significantly (*p* < 0.05) greater reduction in %FHB compared to Proline (Figure 2c), but there was no significant difference in DON (Figure 2d). As there was no interaction between the application of fungicides at T2 and T3, the effect of the two applications was purely additive, resulting in the greatest reduction achieved with the application of APN + PTZ co-formulation at T2 and APN at T3 (92% and 75% control for %FHB and DON, respectively).

## 3. Discussion

There was a wide variation in the levels of FHB and resulting DON for the untreated control plots in each experimental year; this could be explained by differences in weather conditions in each year. In Experiment 1, there was a mild wet spring and the inoculum produced a large number of mature perithecia before flowering. It was then dry during heading and flowering, and consequently the only infection event occurred as a result of mist irrigation at early flowering (mean untreated DON 4.8 mg/kg). In Experiment 2, there was a prolonged dry spell in late spring and very few mature perithecia were visible by early flowering (mean untreated DON 1.4 mg/kg). In Experiment 3, there was a warm, wet spring with high numbers of mature perithecia by wheat head emergence, followed by several rain events during flowering (mean untreated DON 23 mg/kg). One drawback of using spawn inoculum rather than applying spores of *F. graminearum* directly to the flowering crop is the variability in the disease pressure due to seasonal weather impacts on spore maturation and dispersal. However, the advantage is that it creates a much more realistic infection, with spore dispersal occurring over a wide time period.

Results from this study have shown that the new SDHI fungicide, pydiflumetofen in co-formulation with prothioconazole, was significantly better at reducing FHB disease and resulting DON contamination of grain than either pydiflumetofen (A21857B) or prothioconazole (Proline) alone. There are few published studies on the efficacy of pydiflumetofen against FHB and these are either on it a solo product or as a co-formulation with propiconazole (MIRAVIS ACE) [21,22]. These studies have shown pydiflumetofen to be as active, if not slightly better, than triazole fungicides. Previous work and the current study have used different rates of pydiflumetofen; however they have all been compared to equivalent field rates of triazoles. The new co-formulation of pydiflumetofen and prothioconazole (A20944K) has a slightly higher rate of pydiflumetofen compared to MIRAVIS ACE (166 g compared to 150 g pydiflumetofen/ha maximum single field rate), however; the main difference is the higher field rate of a more *Fusarium* active triazole (200 g/ha prothioconazole compared to 125 g/ha propiconazole). 

The first experiment showed that early flowering (GS61) is the most effective timing for control of FHB and resulting DON, with an earlier application at mid-head emergence (GS55) more effective than a later application at late flowering (GS69); this in in agreement with several previous studies [16,21,23,24]. Infection by *F. graminearum* occurs after a rainfall event when temperatures range from 20 to 30 °C with at least 16 h of wetness [25]. Therefore, an application at any growth stage around flowering can be preventative or curative, with an unknown number of days elapsing between infection and fungicide application, depending on the time of infection [26]. Consequently, fungicide efficacy declines with the increasing gap between fungicide application and the infection event, with greater efficacy when applied prior to the infection event [15,26]. If there are multiple infection events during flowering rather than a single event, then fungicide efficacy can again be low. This also means that the optimum head blight spray timing is weather dependent and spraying can be delayed if dry weather is forecast during head emergence and early flowering. However, such flexibility in spray timing will be dictated by the time required for a cereal grower to spray the total area grown and the availability of a spray operator/machine at that time. This study has shown that due to the greater activity of the pydiflumetofen and prothioconazole co-formulation, more effective control can be achieved across a wider time window. This is practically useful when spray operators may have a large area of cereals to spray in a narrow time window and when inclement weather can reduce the number of days suitable to spray within a narrow time window.

A previous study showed that Proline, when applied at the earlier foliar timing of T2 (flag leaf fully emerged, GS39) at a 0.75 maximum single field rate, caused a significant reduction in both FHB (58%) and DON (49%) [17]. The current study showed slightly lower efficacy for Proline at the same timing/application rate (FHB, 52%; DON 43%). For the pydiflumetofen and prothioconazole co-formulation, control was significantly better for DON but not FHB (FHB, 68%; DON 55%) compared to Proline. AscraXpro and Proline were applied at the same field rate of prothioconazole (150 g/ha), with results indicating that the SDHI fungicides—bixafen and fluopyram—did not contribute any additional activity against *F. graminearum*. The lack of any additional control from AscraXpro compared to Proline agrees with previous studies showing low activity of SDHI in general against *F. graminearum* [20,27]. In the UK, the T2 timing (GS39) is a key timing for control of foliar pathogens of wheat and has the largest application area of SDHI fungicides for wheat [28]. Any benefit from a SDHI fungicide applied at T2 to reduce subsequent FHB infection at flowering and DON contamination in harvested wheat grain is therefore an additional reason for the selection of a particular product over another. Another benefit for cereal growers is the availability of an active ingredient from a different chemical class of fungicides that can be used in combination or alternation with triazole fungicides to target *F. graminearum* will reduce selection pressure on the pathogen to develop resistance to either active ingredient.

In conclusion, the availability of a SDHI with good activity against FHB and resulting DON contamination of harvested grain will give growers an additional tool within an IPM program to provide more effective control and greater flexibility of spray application windows and to reduce fungicide resistance selection pressure.

## 4. Materials and Methods

Plots (12 × 2 m) of winter wheat were sown (Crusoe cultivar in Experiment 1 (1918), Extase cultivar in Experiment 2 (2020), and KWS Zyatt cultivar in Experiment 3 (2021)) and maintained per standard agronomic practices at Harper Adams University, apart from the application of *Fusarium* inoculum, application of specific fungicides at earlier growth stages (Table 1), fungicide treatments to target FHB, and mist irrigation.

*Fusarium* spawn inoculum was prepared from five isolates of *F. graminearum* grown on potato dextrose agar (Merck, Darmstadt, Germany) for 5 days. Five mycelial plugs (5 mm diameter) were used to inoculate sterilized oat broth (1 g oat flour in 100 mL deionized water in 250 mL conical flask sealed with aluminum foil). Cultures were incubated for 7 days in an orbital incubator at 120 rpm. One kilogram of oat grains was added to an autoclave bag (78 × 40 cm; VWR International, Lutterworth, UK) with 200 mL deionized water, mixed, folded into a swan neck, and sealed with autoclave tape before autoclaving at 124 °C for one hour. Bags were left 24 h, then re-autoclaved. Each oat broth culture was added to 1.25 L sterile potato dextrose broth (Oxoid Ltd., Basingstoke, UK) and mixed; 100 mL was aliquoted into each bag of autoclaved oats, mixed, folded into a swan neck but not taped, and incubated for three weeks. Every few days, the bags were mixed to avoid the inoculum clumping. All incubations were conducted at room temperature (ca. 18 °C). An equal number of bags of each *F. graminearum* isolate were combined in large bags and well mixed to supply sufficient inoculum for one block of each field experiment. Inoculum was applied to all plots at a rate of 25 g/m^2^ in spring when the wheat was at early stem extension (Zadok’s growth stage 31 [14]). Blanket sprays of fungicides were applied early season to control foliar diseases using a tractor mounted sprayer (Table 1). Fungicide treatments to control FHB were applied to individual plots using a lunchbox knapsack sprayer (Trials Equipment, Braintree, UK) at 3 bar pressure and flat fan (F110-03) nozzles using a volume of 200 L/ha. 

The first experiment was a two-way factorial (timing × fungicide) with an untreated control in a complete randomized block design with four blocks. The timings were mid-head emergence (GS55), early flowering (GS61), and late flowering (GS69). The fungicides were all applied at the maximum single field rate except for APN, which was applied at 2.65 L/ha, which is the maximum field rate for the APN + PTZ co-formulation. Fungicide products included: Proline 275 (ai prothioconazole 275 g/L, 0.72 L/ha, Bayer CropScience Ltd. Cambridge, UK), APN (A21857B; ai pydiflumetofen 62.5 g/L; 2.65 L/ha, Syngenta UK, Cambridge, UK), and APN + PTZ co-formulation (A20944K; ai pydiflumetofen 62.5 g/L plus prothioconazole 75 g/L; 2.65 L/ha, Syngenta UK Ltd.).

The second experiment was a split plot design with the main plot (five sub-plots of 2 × 12 m) treated at full flag leaf emerged (T2, GS39) and the central three sub-plots treated at early flowering (T3, GS61). To avoid spread of inoculum between the main plots, only the central 6 m length of the three central sub-plots were assessed and harvested. This resulted in a minimum 4 m gap between sub-plots treated with different fungicides at the earlier application (T2). Fungicides applied at T2 were at 0.75 of the maximum single field rates and included: untreated, Proline 275 (ai prothioconazole 275 g/L, 0.54 L/ha, Bayer CropScience Ltd.), AscraXpro (ai bixafen 65 g/L, fluopyram 65 g/L and prothioconazole 130 g/L, 1.12 L/ha, Bayer CropScience Ltd.), and APN + PTZ co-formulation (A20944K; ai pydiflumetofen 62.5 g/L plus prothioconazole 75 g/L; 2.65 L/ha, Syngenta). Fungicides applied at T3 were at 0.5 of the maximum single field rates and included: untreated, Proline 275 (ai prothioconazole 275 g/L, 0.54 L/ha, Bayer CropScience Ltd.), and APN (A21857B; ai pydiflumetofen 62.5 g/L; 2.65 L/ha, Syngenta UK Ltd.). All fungicides at T2 were supplemented with Mirror (ai folpet 500 g/L; 1 L/ha). The third experiment was a direct repeat of the second experiment.

After treatment at early flowering (GS61), plots were mist irrigated for 5 days from 5 AM to 10 PM each day for 1 min every 15 min. After misting, the untreated plots were monitored for FHB symptoms (partial senescence of the head) and plots were assessed for FHB once incidence had peaked, but before the wheat heads started to senesce naturally (ca. late milky ripe, GS 77). FHB incidence was assessed by counting the number of infected heads in ten grids (33 × 33 cm) per plot. This was converted to %FHB incidence based on the average number of wheat heads per grid (*n* = 10) for the whole experiment. Once ripe, the crop was harvested with a plot combine (Wintersteiger, Austria); a 1 kg sample was milled (ZM200, Retch; 1 mm screen) and mixed in a tumbler mixer for 5 min before analysis for DON using an AgraQuant^®^ DON ELISA assay (Romer Labs UK Ltd., Runcorn, UK) according to the manufacturer’s instructions.

All statistical analysis was conducted using Genstat (20th Ed, VSN International Ltd. Hemel Hempstead, UK). To obtain normally distributed residuals, the incidence data was logit transformed and the DON data was log10 transformed before using ANOVA. A Fisher LSD test was performed post-hoc to identify differences between individual treatments within a factor and the contrast function was used to compare individual treatments to the untreated control. Data presented are the back-transformed means and percentage control compared to the untreated plots.

## Figures and Tables

**Table 1:** Blanket fungicides applied to each FHB experiment.

Experiment	Timing	Product ^1^	Rate (L/ha)
1	T0 (GS30)	Cherokee	1.3
	T1 (GS32)	Adexar	1
		Bravo	0.5
	T2 (GS39)	Elatus Pro	0.75
		Propov	1
2 and 3	T0 (GS30)	Amistar	0.4
		Ignite	1.5
	T1 (GS32)	Adexar	2
		Mirror	1.5

^1^ Cherokee (ai chlorothalonil 375 g/L, propiconazole 62.5 g/L and cyproconazole 50 g/L, Syngenta); Adexar (ai epoxiconazole 62.5 g/L, and fluxapyroxad 62.5 g/L, BASF); Bravo (ai chlorothalonil 500 g/L, Syngenta); Elatus Pro (ai benzovindiflupyr 100 g/L, Syngenta); Propov (ai epoxiconazole 125 g/L, Syngenta); Amistar (ai azoxystrobin 250 g/L, Syngenta); Ignite (ai epoxiconazole 83 g/L, BASF); Mirror (folpet 500 g/L, Syngenta).

## Data Availability

The data generated in this study are available within the article.
